# Measurements of skeletal muscle mass and power are positively related to a Mediterranean dietary pattern in women

**DOI:** 10.1007/s00198-016-3665-9

**Published:** 2016-07-14

**Authors:** E. Kelaiditi, A. Jennings, C. J. Steves, J. Skinner, A. Cassidy, A. J. MacGregor, A. A. Welch

**Affiliations:** 1 Department of Nutrition, Norwich Medical School, University of East Anglia, Norwich, NR4 7TJ UK; 2 Department of Twin Research and Genetic Epidemiology, King’s College London, London, SE1 7EH UK; 3 Department of Public Health and Primary Care, Norwich Medical School, University of East Anglia, Norwich, NR4 7TJ UK; 4 Department of Medicine, Norwich Medical School, University of East Anglia, Norwich, NR4 7TJ UK

**Keywords:** C-reactive protein, Fat-free mass, Mediterranean diet, Skeletal muscle mass, Skeletal muscle power

## Abstract

**Summary:**

The age-related loss of skeletal muscle and function are risk factors for osteoporosis and fractures. We found that higher adherence to the Mediterranean diet score was significantly associated with greater fat-free mass and leg explosive power suggesting a role for the Mediterranean Diet in prevention of loss of muscle outcomes.

**Introduction:**

The loss of skeletal muscle mass, strength, and function with age are contributing risk factors for the onset of sarcopenia, frailty, osteoporosis, fractures, and mortality. Nutrition may affect the progression and trajectory of these changes in skeletal muscle but the role of the micronutrient-rich Mediterranean diet (MD) has hardly been investigated in relation to these muscle outcomes.

**Methods:**

We examined associations between the MD score (MDS) and FFM% (fat-free mass / weight × 100), FFMI (fat-free mass/height^2^), hand grip strength, and leg explosive power (LEP, watts/kg) in a cross-sectional study in 2570 women aged 18–79 years from the TwinsUK study. Measurements of body composition were made using dual-energy X-ray absorptiometry and dietary intake assessed by a food frequency questionnaire. FFM%, FFMI, grip strength, and LEP were compared across quartiles of the MDS after adjustment for covariates, with CRP measured in a subgroup (*n* = 1658).

**Results:**

Higher adherence to the MDS was positively associated with measurements of muscle outcomes, with significant differences of 1.7 % for FFM% and 9.6 % for LEP (*P* trend <0.001), comparing extreme quartiles of intake, but not with grip strength or CRP concentrations.

**Conclusions:**

For the first time in a northern European population, we have observed significant positive associations between the MDS and FFM% and LEP in healthy women that are potentially clinically relevant, independent of the factors known to influence muscle outcomes. Our findings emphasize the potential role for overall diet quality based on the MD in the prevention of age-related loss of skeletal muscle outcomes.

## Introduction

Sarcopenia (the presence of low skeletal muscle mass and low muscle function) is an increasingly prevalent condition in our ageing populations [1, 2]. Sarcopenia is associated with a number of adverse health outcomes, including physical disability, frailty, and incidence of osteoporosis, falls, and fractures, thus increasing health care costs, and so, it is crucial to identify preventative strategies. Such strategies may be identified in the study of the continuum of the loss of skeletal muscle mass, strength, or function (muscle outcomes) with age using epidemiological techniques [1, 3]. Recent evidence has highlighted that the age-related loss of skeletal muscle mass, strength, or function individually, or in combination, are also associated with an increased risk of osteoporosis and fractures. This may be due to either the potential hormonal or endocrine interactions between skeletal muscle and bone, muscle force-generated mechanical signals, compensation during loss of balance or by acting as a protective barrier to reduce the impact of falls [3, 4]. The mechanisms contributing to the loss of skeletal muscle mass and function with age include chronic low-grade inflammation and oxidative stress, which trigger catabolism and an increase in protein turnover in skeletal muscle [3, 5].

The total quality of the diet measured as food patterns has recently been recognized as important as foods may have “synergistic and cumulative effects on health and disease” beyond the effect of single nutrients [6]. The Mediterranean diet (MD) is characterized by high intakes of fruits and vegetables, legumes, nuts, cereals, and olive oil with low intakes of saturated fat, moderately high intakes of fish, low to moderate intakes of dairy products, low intake of meat, and regular but moderate intake of alcohol [7].

Evidence has been accumulating for the protective effect of the MD for mortality and chronic diseases in particular, cardiovascular disease, hypertension, and cancer [6, 8–10]. Moreover, the recent US dietary guidelines encourage an eating pattern aligned to the MD [6]. In addition to the whole diet, key food components within the MDS have been found to predict chronic disease outcomes, and higher adherence to the MD has been associated with lower circulating inflammatory markers (or cytokines), including C-reactive protein (CRP) [10, 11]. Higher adherence to the MD may also be important for the conservation of skeletal muscle outcomes via the rich concentration of micronutrients associated with higher adherence to the score which may act through their potential anti-inflammatory and anti-oxidant properties (for example vitamins C and E, magnesium and carotenoids) or through a direct role in muscle metabolism and physiology, such as with magnesium and potassium [3, 12–14].

To date, only two cross-sectional and two prospective cohort studies have examined the Mediterranean diet pattern and direct measures of grip or knee strength with only one of these in a population with more than 1000 participants [7, 15–18]. No studies have comprehensively investigated the MD with directly measured skeletal muscle mass, grip strength, muscle function, and low-grade inflammation concurrently in the same population [7, 15–18]. Therefore, the purpose of the present investigation was to evaluate associations between the MD and directly measured muscle outcomes in a cross-sectional study in free-living adult women in a northern European country. We further aimed to investigate the association between the inflammatory marker CRP and the MD, and to determine whether the individual food components of the MDS related independently to muscle outcomes.

## Methods

**Fig. 1:**
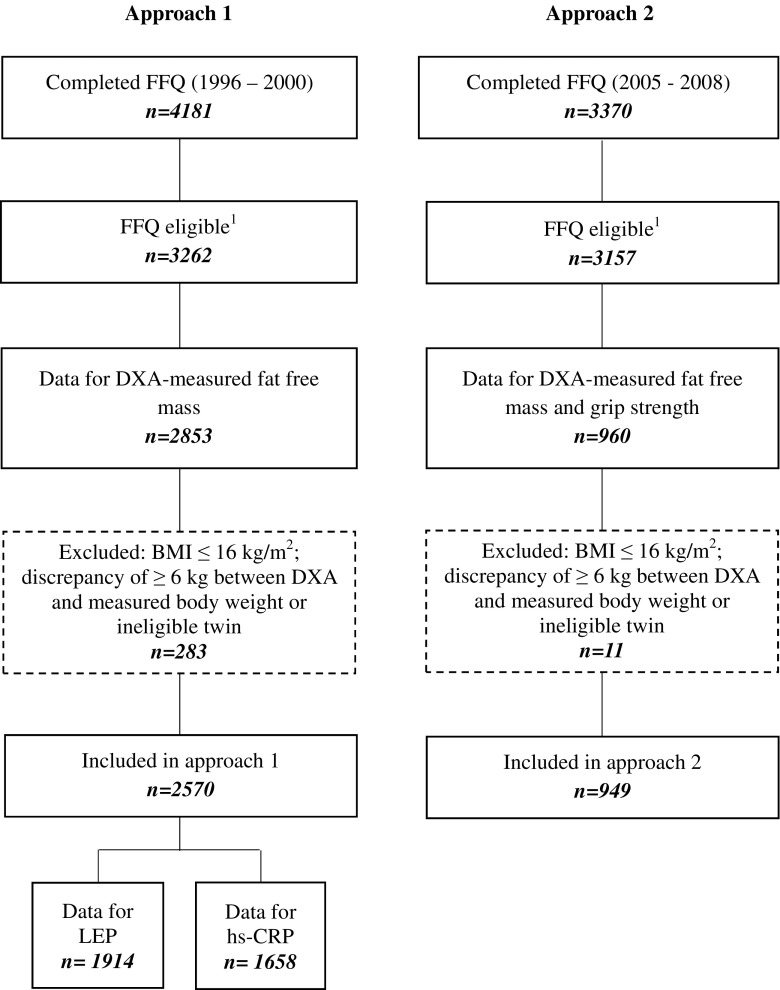
Flowchart of study participants from the TwinUK study. ^1^An eligible FFQ was classified if <10 food items were left blank and the ratio of estimated total energy intake to basal metabolic rate was inside 2 SD of the mean. *FFQ* Food Frequency Questionnaire, *hs-CRP* high-sensitivity C-reactive protein, *LEP* leg explosive power

The women included in this study were from the TwinsUK registry which is an ongoing study of healthy adult twins recruited from the general population who are representative of adult singleton populations in the UK [19, 20]. Data were used from two subsets of this cohort, approach 1 included 2570 women who had completed a food frequency questionnaire (FFQ) and attended for dual-energy X-ray absorptiometry (DXA) measurements between 1996 and 2000 (referred to as the “approach 1” in this manuscript). Within approach 1, there were 1914 individuals with measures of leg explosive power and 1658 individuals who also had measures of high-sensitivity C-reactive protein (hs-CRP). The second group (“approach 2”) consisted of women who had completed an FFQ and had DXA and grip strength measurements measured between 2005 and 2008, see Fig. 1. Ethical approval was obtained from the St. Thomas’s Hospital Research Ethics Committee, and informed consent was acquired from all participants.

### Dietary intake

Participants completed a 131-item validated food frequency questionnaire (FFQ) [21]. Daily nutrient values were calculated using the UK National Nutrient Database [21, 22]. Adherence to MD was evaluated using the MD score (MDS) reported by Trichopoulou et al. [23]. Scoring was based on the intake of the following nine items: vegetables, legumes, fruit and nuts, dairy products, cereals, meat and meat products, fish, alcohol, and the ratio of monounsaturated and polyunsaturated/saturated fat (unsaturated/saturated fat). For food items considered healthy, consumption above the study median received 1 point; all other intakes received 0 point. For dairy products, meat and meat products, consumption below the median received 1 point. For alcohol, women who consumed 5–25 g/day received 1 point; otherwise, the score was 0. The possible scores ranged between 0 and 9, the latter reflecting the maximal adherence. Individuals were excluded from the dietary analyses if answers to >10 food items were left blank or the ratio of estimated total energy intake to the estimated basal metabolic rate fell 2 SDs outside the mean ratio. The dietary intake data was estimated for the appropriate time period for the both approaches. Energy reporting quality (mis-reporting) was calculated using the ratio of reported energy intake (EI) to estimated energy expenditure (EER), the EI/EER ratio, expressed as the percentage of EI to EER, and was included as a covariate for adjustment in the statistical analyses [24].

### Muscle mass, strength, and power

Fat-free mass (FFM) was measured by dual-X-ray absorptiometry (DXA) (Hologic QDR-2000 DXA scanner, Hologic Inc., Waltham, MA, USA). Percentage fat-free mass (FFM%) was calculated as (FFM (kg) / weight (kg) × 100) and fat-free mass index (FFMI) in kg/m^2^ as (FFM (kg) / (height (m)^2^) [25]. Both indices were included in this paper as they scale the proportion of fat-free mass in relation to variation in body size in different ways. Isometric grip strength was assessed using a Jamar hand grip dynamometer (Sammons, Preston, UK) on the dominant arm with reproducibility assessed by repeated measurement on 24 individuals (CV of 11.4 %) [26]. Arm muscle quality was calculated as the ratio of grip strength to mean arm lean mass. Leg explosive power (LEP) was used to measure physical fitness using the Nottingham power rig which measures the force and velocity of muscle contraction principally from the quadriceps [27]. Measurements were normalized for body weight ((LEP (w) / weight (kg)) × mean weight (kg)). LEP has been validated and is highly reliable (reliability coefficient 0.97, coefficient of variation 9.4 %, over 1-week period in adults) [27].

### C-reactive protein

Circulating high-sensitivity C-reactive protein (hs-CRP) was measured by a highly sensitive automated microparticle capture enzyme immunoassay, standardized on the World Health Organization International Reference Standard for CRP immunoassay as previously described [28].

### Covariates

Weight and height were measured with height measured to the nearest 0.5 cm and weight to the nearest 0.1 kg. BMI was calculated as weight in kilograms (kg) divided by height in meters squared (m^2^). Information on lifestyle, medication use, menopausal status, and demographic variables were obtained using a standardized nurse-administered questionnaire. Physical activity was classified as heavy, moderate, or inactive during work, home, and leisure time using a questionnaire that has been strongly correlated with a more in depth assessment of physical activity in this cohort [29].

### Statistical analyses

**Fig. 2:**
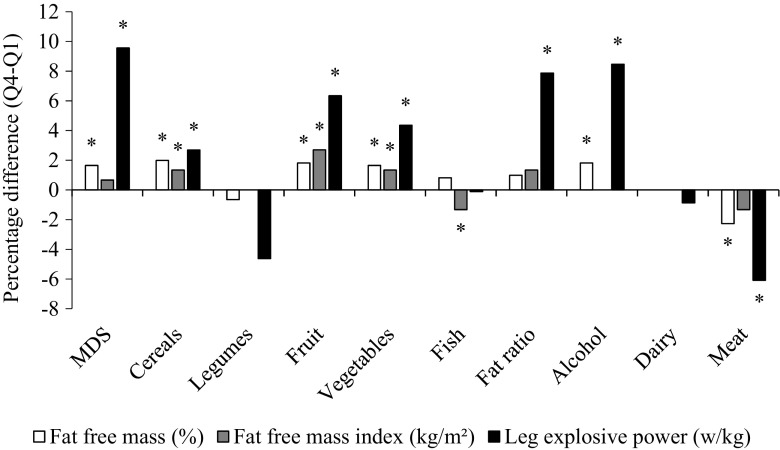
Percentage difference in fat-free mass and leg explosive power between extreme quartiles of the Mediterranean diet score and the individual components. Values are the differences in adjusted means for fat-free mass and leg explosive power in approach 1 between participants in Q4 vs. Q1, expressed as a percentage, *n* = 2570 (fat-free mass) and *n* = 1914 (leg explosive power). Means were adjusted for age, physical activity, smoking status, energy intake and mis-reporting and protein; leg explosive power was additionally adjusted for menopausal status, use of HRT, and height. *P trend < 0.05. *MDS* Mediterranean diet score

Quartiles of the MDS score were calculated and multivariate regression analysis used to calculate statistical trends and the unadjusted and adjusted values for FFM%, FFMI, grip strength, arm muscle quality, LEP, and hs-CRP. In order to check the consistency in data between both approaches, we ran the models for fat-free mass in both the first and second approach. Analyses were conducted for all participants and then stratified by age (<50 and ≥50 years). All models were adjusted for age (years), physical activity (active, moderately active, inactive), smoking status (never, former, current), energy intake (kcal/day), potential mis-reporting of energy intake (EI/EER), and protein intake (g/day) using regression analysis. Protein was included in the models since this has an established role in muscle metabolism, and total energy was included to account for overall differences in energy intake between people of different sizes, an established epidemiological technique [3, 30]. FFMI was additionally adjusted for fat mass (kg), and arm muscle quality and LEP were also adjusted for menopausal status (premenopausal/ postmenopausal), use of HRT (yes/no), and height (m). Hs-CRP was also adjusted for BMI, use of anti-inflammatory medications (yes/no) and HRT (yes/no). Values for hs-CRP were skewed, and therefore, natural log-transformed values were used for the analyses. Values in the text are means ± SE and for hs-CRP geometric mean (95 % CI). The percentage difference between Q1 and Q4 was calculated for FFM% and LEP as (Q4-Q1 / Q1) × 100. For the analysis shown in Fig. 2, the percentage difference in FFM%, FFMI, and LEP between extreme quartiles of the MDS was calculated as (Q4-Q1 / Q1) × 100. A *P* value <0.05 was considered statistically significant. We tested whether age moderated the association between MDS and muscle outcomes by including an interaction term (age × MDS) in the models. The correlations between the individual components of the MDS were calculated.

In a further analysis to explore these associations independently of genetics, we identified twin pairs discordant for MDS, defined as a within-pair difference in MDS of at least 4 points. We tested the intra-pair differences in muscle outcomes and MDS adjusted for smoking, physical activity, and BMI in regression models.

All analyses were performed with Stata statistical software version 11.0 (Stata Corp, College Station, TX) using the participants as individuals and included the robust cluster regression option in STATA to account for clustering within twin pairs.

## Results

The characteristics of the participants in the two subsets are presented in Table 1. The women in the approach 2 were older than those in approach 1, which was expected given the timing of the measurements. The women in approach 2 also had a higher BMI, reported less physical activity, and were less likely to smoke. There were no apparent differences in dietary intake between the approaches.

**Table 1:** Characteristics and dietary intakes of females aged 18–79 years

Characteristics	Approach 1 (*n* = 2570)	Approach 2 (*n* = 949)
Age (years)	48.3 ± 12.7	59.1 ± 9.3
BMI (kg/m^2^)	24.9 ± 4.1	26.5 ± 4.7
Fat mass (kg)	22.7 ± 7.9	–
Fat-free mass (%)	61.1 ± 6.5	–
Fat-free mass index (kg/m^2^)	15.0 ± 1.7	–
Hand grip strength (kg)	–	28.8 ± 5.9
Arm muscle quality (kg/kg)	–	13.4 ± 2.8
Leg explosive power^a^ (watts/kg)	90.9 ± 36.5	–
hs-CRP^b^ (mg/L)	2.49 ± 2.5	–
Mediterranean diet score (points)	4.3 ± 1.8	4.3 ± 1.8
Cereals (g/day)	207 ± 103	
Legumes (g/day)	23.3 ± 26.3	
Fruit and nuts (g/day)	232 ± 195	
Vegetables (g/day)	279 ± 157	
Fish (g/day)	35.9 ± 27.5	
Fat ratio (unsaturated/saturated fat)	1.5 ± 0.4	
Alcohol (g/day)	10.0 ± 13.5	
Dairy products (g/day)	421 ± 197	
Meat and meat products (g/day)	86.8 48.6	
Energy intake (kcal/day)	1979 ± 524	1911 ± 635
Protein (g)	81.3 ± 21.6	83.3 ± 27.2
Mis-reporting (EI/EER, %)	87.4 ± 24.6	89.2 ± 33.7
Physical activity		
Active (%)	24.2 % (622)	26.0 % (247)
Moderately active (%)	53.9 % (1385)	34.5 % (327)
Inactive (%)	21.9 % (563)	39.5 % (375)
Smoking status (current, %)	18.2 % (468)	9.7 % (92)
Anti-inflammatory medication^b^ (yes, %)	6.2 % (102)	–
Menopausal status^a^ (postmenopausal, %)	42.1 % (806)	89.8 % (852)
Hormone replacement therapy^a^ (yes, %)	14.9 % (285)	9.5 % (90)

Values are mean (SD) or % (*n*=), *n* = 2570. Fat ratio—unsaturated/saturated fat (total polyunsaturated + total monounsaturated fat/saturated fat). *EI/EER* ratio of reported energy intake to estimated energy requirements. Arm muscle quality—ratio of grip strength to arm muscle mass (kg/kg)

^a^Values for a subset of 1914 participants in the fat-free mass group

^b^Values for a subset of 1658 participants in the fat-free mass group

In multivariable analyses, women in the highest quartile of the MDS had significantly higher fat-free mass and LEP, compared to those in the lowest quartile (Table 2). Specifically, FFM% was 1.0 % higher (±0.3, *P* trend <0.001) (equivalent to 1.7 % higher) and LEP was 8.3 w/kg higher (±2.2, *P* trend <0.001) (equivalent to 9.6 % higher) when comparing extreme quartiles of MDS. We also found these associations in approach 2 finding a difference of 1.4 ± 0.5 % for FFM% (*P* = 0.009) and 0.1 ± 0.1 kg/m^2^ for FFMI (*P* = 0.72), data not shown. We observed no significant interactions in approach 1 for FFM%, FFMI, or LEP (all *P* < 0.05) with stratification for age, but as it is important to understand the relationship between diet and skeletal muscle outcomes in younger as well as older ages, the stratified analyses were retained. When stratified by age, differences in FFM% and LEP were greater for women aged over 50 years, compared to those aged under 50 years (Table 3). For LEP comparing quartile 4 vs. quartile 1 of the MDS, there was a 13 % difference for women aged over 50 years (9.5 w/kg ± 3.0, *P* trend = 0.01) compared to an 8 % difference in the younger age group (7.4 w/kg ± 3.2, *P* trend = 0.01), a 5 % difference between the two age groups. There were no significant differences in FFMI, grip strength, arm muscle quality, or levels of hs-CRP when all women were considered together, although FFMI was significantly higher in the highest compared with the lowest MDS in the group aged less than 50 y (0.2 kg/m^2^ ± 0.1, *P* trend = 0.028) (Table 3). In the further analysis to explore the associations independent of genetics in twins, 27 twin pairs were identified as discordant for MDS in approach 1 (data for LEP was available for 17 of these pairs). Mean MDS was 2.3 ± 1.3 in the lower intake twins and 6.8 ± 1.0 in the higher intake twins. The adjusted regression coefficients of the intra-pair difference in muscle outcomes and MDS were as follows: FFM% 1.4 ± 1.1, *P* = 0.20; FFMI −0.3 ± 0.3, *P* = 0.33; and LEP 4.4 ± 11.6, *P* = 0.71.

**Table 2:** Measures of muscle mass, muscle strength, and inflammation by quartile of Mediterranean diet score in 2570 females aged 18–79 years

	Model	Q1	Q2	Q3	Q4	Q4-Q1	*P* trend
Mediterranean diet score (points)	–	0–3	4	5	6–9	–	–
Fat-free mass (%)	*n*=	897	538	461	674	–	–
	1	60.9 ± 0.3	60.6 ± 0.3	61.3 ± 0.3	61.6 ± 0.3	0.7 ± 0.4	0.021
	2	60.7 ± 0.2	60.6 ± 0.3	61.6 ± 0.3	61.6 ± 0.2	0.9 ± 0.3	<0.001
	3	60.7 ± 0.2	60.6 ± 0.3	61.6 ± 0.3	61.7 ± 0.2	1.0 ± 0.3	<0.001
Fat-free mass index (kg/m^2^)	*n*=	897	538	461	674	–	–
	1	14.9 ± 0.1	15.0 ± 0.1	15.2 ± 0.1	15.1 ± 0.1	0.1 ± 0.1	0.050
	2	15.0 ± 0.1	15.0 ± 0.1	15.1 ± 0.1	15.1 ± 0.1	0.1 ± 0.1	0.076
	3	15.0 ± 0.1	15.0 ± 0.1	15.1 ± 0.1	15.1 ± 0.1	0.1 ± 0.1	0.086
Grip strength^a^ (kg)	*n*=	303	214	188	244	–	–
	1	28.6 ± 0.4	28.2 ± 0.5	28.8 ± 0.4	29.4 ± 0.4	0.8 ± 0.5	0.470
	2	28.9 ± 0.3	28.6 ± 0.4	28.8 ± 0.4	28.7 ± 0.3	−0.1 ± 0.5	0.855
	3	28.8 ± 0.3	28.6 ± 0.4	28.8 ± 0.4	28.7 ± 0.3	−0.1 ± 0.5	0.855
Arm muscle quality^a^ (kg/kg)	*n*=	303	214	188	244	–	–
	1	13.3 ± 0.2	13.1 ± 0.2	13.6 ± 0.2	13.7 ± 0.2	0.4 ± 0.2	0.077
	2	13.4 ± 0.1	13.2 ± 0.2	13.5 ± 0.2	13.5 ± 0.2	0.1 ± 0.2	0.472
	3	13.4 ± 0.1	13.2 ± 0.2	13.5 ± 0.2	13.5 ± 0.2	0.1 ± 0.2	0.472
Leg explosive power^b^ (watts/kg)	*n*=	662	410	340	502	–	–
	1	87.4 ± 1.5	90.3 ± 1.8	92.6 ± 2.0	94.7 ± 1.8	7.3 ± 2.3	0.001
	2	86.8 ± 1.4	90.8 ± 1.8	92.5 ± 1.9	95.0 ± 1.7	8.2 ± 2.2	<0.001
	3	86.8 ± 1.4	90.7 ± 1.8	92.7 ± 1.9	95.1 ± 1.7	8.3 ± 2.2	<0.001
C-reactive protein^c^ (mg/L)	*n*=	497	359	315	487	–	–
	1	1.6 (1.5, 1.8)	1.6 (1.4, 1.8)	1.6 (1.5, 1.8)	1.6 (1.4, 1.7)	–	0.644
	2	1.6 (1.5, 1.8)	1.6 (1.4, 1.7)	1.6 (1.5, 1.8)	1.6 (1.5, 1.7)	–	0.879
	3	1.6 (1.5, 1.8)	1.6 (1.4, 1.7)	1.6 (1.4, 1.8)	1.6 (1.5, 1.7)	–	0.842

Values are mean ± SE, *n* = 2570. Model 1 was unadjusted. Model 2 was adjusted for age, physical activity, smoking status, energy intake, and mis-reporting, and fat-free mass index was additionally adjusted for fat mass. Model 3 was adjusted for the variables in model 2, plus protein intake. *P* trend values were calculated using ANCOVA

^a^Analysis for approach 2. Values are mean ± SE, *n* = 949. Model 1 was unadjusted. Model 2 was adjusted for age, physical activity, smoking status, energy intake, mis-reporting, menopausal status, use of HRT, and height. Model 3 was adjusted for the variables in model 2, plus protein intake

^b^Subset analysis for approach 1. Values are mean ± SE, *n* = 1914. Model 1 was unadjusted. Model 2 was adjusted for age, physical activity, smoking status, energy intake, mis-reporting, menopausal status, use of HRT, and height. Model 3 was adjusted for the variables in model 2, plus protein intake

^c^Subset analysis for approach 1. Values are geometric mean (95 % CI), *n* = 1658. Model 1 was unadjusted. Model 2 was adjusted for age, BMI, physical activity, smoking status, energy intake, mis-reporting, and use of anti-inflammatory medication and HRT. Model 3 was adjusted for the variables in model 2, plus protein intake

**Table 3:** Measures of muscle mass, muscle strength, and inflammation by quartile of Mediterranean diet score in 2570 females stratified by age

	Age (years)	Number	Q1	Q2	Q3	Q4	Q4-Q1	*P* trend
Mediterranean diet score (points)	–		0–3	4	5	6–9	–	–
Fat-free mass (%)	<50	1264	63.0 ± 0.3	62.7 ± 0.3	63.8 ± 0.4	63.9 ± 0.3	0.9 ± 0.4	0.012
	≥50	1306	58.5 ± 0.3	58.5 ± 0.4	59.4 ± 0.4	59.5 ± 0.3	1.0 ± 0.4	0.008
Fat-free mass index (kg/m^2^)	<50	1264	15.0 ± 0.1	15.0 ± 0.1	15.2 ± 0.1	15.2 ± 0.1	0.2 ± 0.1	0.028
	≥50	1306	14.9 ± 0.1	15.0 ± 0.1	15.1 ± 0.1	15.0 ± 0.1	0.1 ± 0.1	0.539
Grip strength^a^ (kg)	<50	132	32.5 ± 0.7	34.1 ± 0.9	31.9 ± 1.5	32.9 ± 0.8	0.3 ± 1.0	0.912
	≥50	817	28.3 ± 0.3	27.7 ± 0.4	28.2 ± 0.4	28.1 ± 0.4	−0.1 ± 0.5	0.975
Arm muscle quality^a^ (kg/kg)	<50	132	14.9 ± 0.3	14.5 ± 0.4	14.5 ± 0.7	14.5 ± 0.4	0.4 ± 0.5	0.484
	≥50	817	13.2 ± 0.2	13.0 ± 0.2	13.3 ± 0.2	13.4 ± 0.2	0.1 ± 0.2	0.324
Leg explosive power^b^ (watts/kg)	<50	1074	97.1 ± 2.0	96.3 ± 2.4	102.4 ± 2.6	104.5 ± 2.5	7.4 ± 3.2	0.010
	≥50	840	73.9 ± 1.9	83.1 ± 2.7	79.5 ± 2.7	83.3 ± 2.3	9.5 ± 3.0	0.005
C-reactive protein^c^ (mg/L)	<50	734	1.5 (1.3, 1.7)	1.2 (1.1, 1.4)	1.4 (1.1, 1.6)	1.3 (1.2, 1.5)	–	0.512
	≥50	924	1.8 (1.6, 2.0)	1.9 (1.7, 2.2)	1.9 (1.6, 2.1)	1.8 (1.6, 2.0)	–	0.723

Values are mean ± SE, *n* = 1295 < 50 years and *n* = 1306 ≥ 50 years. Values were adjusted for age, physical activity, smoking status, energy intake, mis-reporting, protein intake, and fat-free mass index was additionally adjusted for fat mass. *P* trend values were calculated using ANCOVA

^a^Analysis for the approach 2 group. Values are mean ± SE, *n* = 132 < 50 years and *n* = 817 ≥ 50 years. Values were adjusted for age, physical activity, smoking status, energy intake, mis-reporting, protein intake, menopausal status, use of HRT, and height

^b^Subset analysis for the approach 1. Values are mean ± SE, *n* = 1074 < 50 years and *n* = 840 ≥ 50 years. Values were adjusted for age, physical activity, smoking status, energy intake, protein intake, mis-reporting, menopausal status, use of HRT, and height

^c^Subset analysis for approach 1. Values are mean ± SE, n = 734 < 50 years and n = 924 ≥ 50 years. Values were adjusted for age, BMI, physical activity, smoking status, energy intake, mis-reporting, protein intake, use of anti-inflammatory medication and HRT

To elucidate the specific constituents of the Mediterranean diet that might be responsible for these associations, intakes of the individual food components of the MDS were examined in relation to FFM%, FFMI, and LEP (Fig. 2). In the correlation analysis between the Mediterranean diet and food groups the correlations were weak with the highest correlation being 0.4 between the ratio of unsaturated to saturated fat and vegetable intake and with the correlation between vegetable and fruit intake being of a similar scale. Of the nine food categories used to determine the MDS, cereals, and fruits and vegetables showed positive, significant adjusted trends with FFM%, FFMI, and LEP. In addition, intakes of alcohol were positively associated with FFM% and LEP, meat was negatively related, and the ratio of fats was significantly, and positively, related to LEP only. For arm muscle quality and hs-CRP, fruit intake was significantly associated with both outcomes (arm muscle quality Q4-Q1 3.7 %, *P* trend = 0.043; hs-CRP Q4-Q1 12.6 %, *P* trend 0.038, data not shown) and alcohol intake with arm muscle quality only (Q4-Q1 3.0 %, *P* trend = 0.012). No significant associations were observed between intakes of legumes, fish, or dairy products for any of the outcome measures. However, the overall association with the MDS was stronger than for any of the individual components, Fig. 2.

## Discussion

In this large-scale cross-sectional study of 18–79-year-old women, we found significant positive associations between diet quality measured by the Mediterranean diet and indices of fat-free mass and LEP. The differences found between higher and lower adherence to the MDS were 1.7 % for FFM% and 9.6 % for LEP. These associations remained after adjustment for age, physical activity, smoking status, energy and protein intake and mis-reporting and for LEP, use of HRT and body weight, indicating that the findings were independent of these covariates. Within our discordance analysis, to ascertain potential gene influences, we found no associations between those with differences in the MDS and skeletal muscle outcomes which may indicate that genetics could underly the relationships found here. Nevertheless, the number of twin pairs with discordance for intake was small and our analyses may have been underpowered to detect an effect. However, our findings may have clinical relevance. When comparing the scale of our findings with the known loss of LEP with age (of 3.5 % per year in a study of older women), the relationship with the MDS of 9.6 % was 3.5 times that of the loss per year [31]. For FFM%, using a conservative estimate from a longitudinal study of loss of 0.7 % FFM per year, our finding of 1.7 % between extreme quartiles of the MDS is 2.4 times that of the measured loss of FFM per year [3, 32, 33]. To our knowledge, this is the first representative population study to investigate associations between the MDS in a northern European population and risk factors for sarcopenia and frailty, as previous studies have all focused on older adults, although it is known that age-related declines in muscle mass occur as a continuum in younger as well as older adults [7, 15–17, 34, 35]. Furthermore, when stratified by age the associations with the MD in population for LEP were greater for women aged over 50 years, compared to those aged under 50 years, with the between quartile differences being 5 % greater in older than in younger women. Differences in FFM% were also greater but for FFMI the difference across quantiles was significant only in women aged less than 50 years. Interestingly, we observed no associations between the MDS and arm muscle quality or C-reactive protein in our study.

The lack of association between the MDS and grip strength in our study is similar to four previous studies that directly measured muscle strength and the MD, all in older populations, finding no association or a significant positive association only in unadjusted analyses [7, 15–18]. The only other study that related skeletal muscle mass (as appendicular skeletal muscle mass/height^2^) to the MDS (defined a posteriori) found no association between skeletal muscle mass or of sarcopenia in Iranian men and women [34]. Also, a recent study in older Chinese men and women found no association between prevalent or incident sarcopenia and the MDS during 4 years of follow-up [36]. Although our findings are in line with previous studies, our lack of association between the MDS and grip strength may be because age is such a strong determinant in cross-sectional studies, and the mean hand grip strength in our study was higher than that found in a previous study of older women in the UK [37]. However, our analyses to determine consistency between the approaches in this study for skeletal muscle outcomes demonstrated the same associations between indices of FFM and the MDS in women over time.

Our further investigation of the food categories that contributed to the MDS and the muscle outcomes of FFM%, FFMI, and LEP found that cereals, fruits, and vegetables were positively and significantly related. Alcohol intake was also positively related to FFM% and LEP. However, the ratio of unsaturated to saturated fats (the total of monounsaturated and polyunsaturated fat to saturated fat) related to LEP only, although we previously found a relationship between FFMI and the P/S (total polyunsaturated fat to saturated fat) ratio in this population [38]. Surprisingly, we also found a significant inverse association between meat consumption and FFM% and LEP that was independent of protein intake. The explanation for this is unknown although we previously reported an inverse relationship between a greater dietary acid-base load and indices of skeletal muscle mass [39, 40]. Meat is a major contributor to the dietary acid-base load, with fruits and vegetables acting to alkalinize dietary intake [39, 40]. We found no independent association with arm muscle quality and total fish intake in comparison with the one other study that found a positive association with grip strength and fatty fish consumption in older women living in the UK [37]. However, importantly, the association with the MDS which characterizes a combined approach to healthy eating was greater than any of its individual components.

We anticipated, on the basis of previous research, that the MDS may influence inflammation and therefore be a mechanism that might potentially explain the link between muscle outcomes and the MDS; however, we did not find a relationship in this study [14]. This may be for a number of reasons including the fact that the mean concentrations of circulating CRP were low in our cohort, due to the age range, or that more than one measure of an inflammatory cytokine would better capture inflammatory status. It may also be that the distribution of potentially anti-oxidant or anti-inflammatory nutrients and foods associated with the MD, within a northern European country, may not be as great as in studies of the MD performed within Mediterranean countries [41, 42]. Alternatively, the benefits of the MDS in a European population on skeletal muscle outcomes may be due to other mechanisms such as effects on the gut microbiota, which has been linked to the MD in human studies, and to markers of muscle atrophy in animal studies [43]. Further work is warranted using alternative markers of inflammation.

The strengths of this study include the large sample size and the wide age range of participants, as most previous studies on sarcopenia, or the muscle outcomes associated with sarcopenia, focused only on older individuals. Moreover, this was the first study that compared the MDS with directly measured indices of skeletal muscle mass, strength, and function in a large cohort. The FFQ used in the current study was previously validated for protein, vitamin C, potassium, sodium, and n-3 PUFAs and has been shown to rank episodically consumed foods, such as fish, equally well when compared with other dietary methods [30, 44, 45]. The observed findings relate to women and further work is needed to investigate if these findings are replicated in a male population of the same country or in populations from different ethnic backgrounds.

Our study also has limitations, which warrant discussion. The cross-sectional nature of the study limits causal inference between the MDS and muscle outcomes. Although low-grade inflammation was measured using circulating CRP, the lower concentrations found in this population may have been insufficient to relate to an effect of overall diet. Higher adherence to the MDS may also be an indicator of a healthy lifestyle, and even though our analyses were adjusted for all the known lifestyle factors that impact on muscle outcomes, residual confounding cannot be ruled out.

In conclusion, we found that diet quality assessed by the predefined MDS is positively associated with indices of skeletal muscle mass and function in women of different ages. Moreover, our findings may have clinical relevance as the scale of associations found were 2.4 and 3.5 times the measured loss of FFM and LEP per year, respectively, even after accounting for covariates. These novel findings suggest that for adult women a healthier dietary pattern of the MDS specifically of higher intakes in fruit and vegetables, cereal foods, the unsaturated to saturated fat ratio and alcohol along with lower intakes of meats, may be important in reducing the loss of skeletal muscle mass strength and function with age. These findings provide important information in developing and planning potential dietary intervention trials for the prevention of sarcopenia. From a public health perspective, our findings support the current healthy eating guidelines to consume a diet high in plant and whole grain cereal foods that is lower in saturated fat intakes and suggest that a healthy eating pattern characterized by the MDS could be beneficial for skeletal muscle outcomes.

## References

[1] Cruz-Jentoft AJ, Baeyens JP, Bauer JM, Boirie Y, Cederholm T, Landi F (2010). Sarcopenia: European consensus on definition and diagnosis: report of the European Working Group on Sarcopenia in Older People. Age Ageing.

[2] Patel HP, Syddall HE, Jameson K, Robinson S, Denison H, Roberts HC (2013). Prevalence of sarcopenia in community-dwelling older people in the UK using the European Working Group on Sarcopenia in Older People (EWGSOP) definition: findings from the Hertfordshire Cohort Study (HCS). Age Ageing.

[3] Welch AA (2014). Nutritional influences on age-related skeletal muscle loss. Proc Nut Soc.

[4] DiGirolamo DJ, Kiel DP, Esser KA (2013). Bone and skeletal muscle: neighbors with close ties. J Bone Miner Res.

[5] Roubenoff R, Hughes VA (2000). Sarcopenia. J Gerontol Ser A Biol Med Sci.

[6] USDA, Dietary Guidelines for Americans 2015–2020, t.O.o.D.P.a.H. Promotion, Editor. 2016, the Office of Disease Prevention and Health Promotion: US

[7] Milaneschi Y, Bandinelli S, Corsi AM, Lauretani F, Paolisso G, Dominguez LJ (2011). Mediterranean diet and mobility decline in older persons. Exp Gerontol.

[8] Liese AD, Krebs-Smith SM, Subar AF, George SM, Harmon BE, Neuhouser ML (2015). The Dietary Patterns Methods Project: synthesis of findings across cohorts and relevance to dietary guidance. J Nutr.

[9] Gotsis E, Anagnostis P, Mariolis A, Vlachou A, Katsiki N, Karagiannis A (2015). Health benefits of the Mediterranean Diet: an update of research over the last 5 years. Angiology.

[10] Ostan R, Lanzarini C, Pini E, Scurti M, Vianello D, Bertarelli C (2015). Inflammaging and cancer: a challenge for the Mediterranean diet. Nutrients.

[11] Schwingshackl L, Hoffmann G (2014). Mediterranean dietary pattern, inflammation and endothelial function: a systematic review and meta-analysis of intervention trials. Nutr Metab Cardiovasc Dis.

[12] Mangge H, Becker K, Fuchs D, Gostner JM (2014). Antioxidants, inflammation and cardiovascular disease. World J Cardiol.

[13] Denison HJ, Cooper C, Sayer AA, Robinson SM (2015). Prevention and optimal management of sarcopenia: a review of combined exercise and nutrition interventions to improve muscle outcomes in older people. Clin Interv Aging.

[14] Welch, A.A., E. Kelaiditi, A. Jennings, C.J. Steves, T.D. Spector, and A. MacGregor (2015) Dietary magnesium is positively associated with skeletal muscle power and indices of muscle mass and may attenuate the association between circulating C-reactive protein and muscle mass in women. J Bone Miner Res 10.1002/jbmr.2692 26288012

[15] Bollwein J, Diekmann R, Kaiser MJ, Bauer JM, Uter W, Sieber CC (2013). Dietary quality is related to frailty in community-dwelling older adults. J Gerontol A Biol Sci Med Sci.

[16] Talegawkar SA, Bandinelli S, Bandeen-Roche K, Chen P, Milaneschi Y, Tanaka T (2012). A higher adherence to a Mediterranean-style diet is inversely associated with the development of frailty in community-dwelling elderly men and women. J Nutr.

[17] Zbeida M, Goldsmith R, Shimony T, Vardi H, Naggan L, Shahar DR (2014). Mediterranean diet and functional indicators among older adults in non-Mediterranean and Mediterranean countries. J Nutr Health Aging.

[18] Fougere, B., S. Mazzuco, P. Spagnolo, S. Guyonnet, B. Vellas, M. Cesari, et al. (2015) The association between the Mediterranean-style dietary pattern score and physical activity performance: results from the Trelong Study. J Nutr Health Aging 10.1007/s12603-015-0588-7 PMC12879822 26999242

[19] Teucher B, Skinner J, Skidmore PM, Cassidy A, Fairweather-Tait SJ, Hooper L (2007). Dietary patterns and heritability of food choice in a UK female twin cohort. Twin Res Hum Genet.

[20] Andrew T, Hart DJ, Snieder H, de Lange M, Spector TD, MacGregor AJ (2001). Are twins and singletons comparable? A study of disease-related and lifestyle characteristics in adult women. Twin Res.

[21] Welch AA, Luben R, Khaw KT, Bingham SA (2005). The CAFE computer program for nutritional analysis of the EPIC-Norfolk food frequency questionnaire and identification of extreme nutrient values. J Hum Nutr Diet.

[22] McCance, R.A., E.M. Widdowson, B. Holland, A.A. Welch, D.H. Buss, F. Great Britain. Ministry of Agriculture, et al., McCance and Widdowson’s the composition of foods. 5th rev & extended ed. 1991: Royal Society of Chemistry

[23] Trichopoulou A, Costacou T, Bamia C, Trichopoulos D (2003). Adherence to a Mediterranean diet and survival in a Greek population. N Engl J Med.

[24] Otten JJ, Hellwig JP, Meyers LD (2006). DRI, dietary reference intakes: the essential guide to nutrient requirements.

[25] Kyle UG, Schutz Y, Dupertuis YM, Pichard C (2003). Body composition interpretation. Contributions of the fat-free mass index and the body fat mass index. Nutrition.

[26] Arden NK, Spector TD (1997). Genetic influences on muscle strength, lean body mass, and bone mineral density: a twin study. J Bone Miner Res.

[27] Bassey EJ, Short AH (1990). A new method for measuring power output in a single leg extension: feasibility, reliability and validity. Eur J Appl Physiol Occup Physiol.

[28] MacGregor AJ, Gallimore JR, Spector TD, Pepys MB (2004). Genetic effects on baseline values of C-reactive protein and serum amyloid a protein: a comparison of monozygotic and dizygotic twins. Clin Chem.

[29] Cherkas LF, Hunkin JL, Kato BS, Richards JB, Gardner JP, Surdulescu GL (2008). The association between physical activity in leisure time and leukocyte telomere length. Arch Intern Med.

[30] Willett W (2013). Nutritional Epidemiology (3rd Edition).

[31] Skelton DA, Greig CA, Davies JM, Young A (1994). Strength, power and related functional ability of healthy people aged 65–89 years. Age Ageing.

[32] Mitchell WK, Williams J, Atherton P, Larvin M, Lund J, Narici M (2012). Sarcopenia, dynapenia, and the impact of advancing age on human skeletal muscle size and strength; a quantitative review. Front Physiol.

[33] Koster A, Ding J, Stenholm S, Caserotti P, Houston DK, Nicklas BJ (2011). Does the amount of fat mass predict age-related loss of lean mass, muscle strength, and muscle quality in older adults?. J Gerontol Ser A, Biol Sci Med Sci.

[34] Hashemi R, Motlagh AD, Heshmat R, Esmaillzadeh A, Payab M, Yousefinia M (2015). Diet and its relationship to sarcopenia in community dwelling Iranian elderly: a cross sectional study. Nutrition.

[35] Baumgartner RN (2000). Body composition in healthy aging. Ann N Y Acad Sci.

[36] Chan R, Leung J, Woo J (2016). A prospective cohort study to examine the association between dietary patterns and sarcopenia in Chinese community-dwelling older people in Hong Kong. J Am Med Dir Assoc.

[37] Robinson SM, Jameson KA, Batelaan SF, Martin HJ, Syddall HE, Dennison EM (2008). Diet and its relationship with grip strength in community-dwelling older men and women: the Hertfordshire cohort study. J Am Geriatr Soc.

[38] Welch AA, Macgregor AJ, Minihane AM, Skinner J, Valdes AA, Spector TD (2014). Dietary fat and Fatty Acid profile are associated with indices of skeletal muscle mass in women aged 18–79 years. J Nutr.

[39] Welch, A.A., A.J. Macgregor, J. Skinner, T.D. Spector, A. Moayyeri, and A. Cassidy (2012) A higher alkaline dietary load is associated with greater indexes of skeletal muscle mass in women. Osteoporos Int 10.1007/s00198-012-2203-7 23152092

[40] Welch AA, Mulligan A, Bingham SA, Khaw KT (2008). Urine pH is an indicator of dietary acid–base load, fruit and vegetables and meat intakes: results from the European Prospective Investigation into Cancer and Nutrition (EPIC)-Norfolk population study. Br J Nutr.

[41] Freisling H, Fahey MT, Moskal A, Ocke MC, Ferrari P, Jenab M (2010). Region-specific nutrient intake patterns exhibit a geographical gradient within and between European countries. J Nutr.

[42] Trichopoulou A, Martinez-Gonzalez MA, Tong TY, Forouhi NG, Khandelwal S, Prabhakaran D (2014). Definitions and potential health benefits of the Mediterranean diet: views from experts around the world. BMC Med.

[43] Haro, C., S. Garcia-Carpintero, J.F. Alcala-Diaz, F. Gomez-Delgado, J. Delgado-Lista, P. Perez-Martinez, et al. (2015) The gut microbial community in metabolic syndrome patients is modified by diet. J Nutr Biochem 10.1016/j.jnutbio.2015.08.011 26376027

[44] Welch AA, Bingham SA, Ive J, Friesen MD, Wareham NJ, Riboli E (2006). Dietary fish intake and plasma phospholipid n-3 polyunsaturated fatty acid concentrations in men and women in the European Prospective Investigation into Cancer-Norfolk United Kingdom cohort. Am J Clin Nutr.

[45] McKeown NM, Day NE, Welch AA, Runswick SA, Luben RN, Mulligan AA (2001). Use of biological markers to validate self-reported dietary intake in a random sample of the European Prospective Investigation into Cancer United Kingdom Norfolk cohort. Am J Clin Nutr.

